# Psychoactive substance use among first-year students in a Botswana University: pattern and demographic correlates

**DOI:** 10.1186/s12888-018-1844-2

**Published:** 2018-08-31

**Authors:** Anthony A. Olashore, Olorunfemi Ogunwobi, Eden Totego, Philip R. Opondo

**Affiliations:** 1 Department of Psychiatry, University of Botswana Medical School, Private Bag, 00712 Gaborone, Botswana; 2 Department of Psychiatry, Bowen University Teaching Hospital, Ogbomosho, Nigeria; 3 University of Botswana Medical School, Gaborone, Botswana

**Keywords:** Substance use, Risk factors, Psychological distress, University students, Botswana

## Abstract

**Background:**

Substance use amongst university students is a recognized problem worldwide. Few studies have been carried out in this group in Botswana. These studies have been mostly limited to the use of alcohol and tobacco. Therefore, this study was designed to investigate the pattern of general substance use, its association with psychological distress and common socio-demographic factors among first-year undergraduates in a Botswana University.

**Methods:**

A total of 401 students were interviewed using a *modified W.H.O. student drug use questionnaire* and the 12 item *General Health Questionnaire* (GHQ12) to assess the pattern of psychoactive substance use and its relationship with psychological distress amongst university students in Botswana.

**Results:**

Alcohol was the most (31.9%) commonly used psychoactive substance. Age of debut for most psychoactive substances was between the ages of 15–18 years. Current use of alcohol (*p* = 0.045), amphetamine-type stimulants (*p* = 0.004) and benzodiazepines (*p* = 0.021) were associated with significant psychological distress. A positive relationship was observed between low participation in religious activities and substance use (OR = 4.63, 95%CI: 2.03–10.51), while a negative association was observed between not having a friend who uses drugs and substance use (OR = 0.44, 95%CI: 0.19–0.99).

**Conclusions:**

There is a significant substance abuse problem in the undergraduate population in Botswana. Our findings followed the global trend, with alcohol being the most commonly used substance. Religious participation demonstrates potential to be one of the solutions to this problem, but how to harness its seemingly protective influences is a field for further study.

## Background

For several centuries, psychoactive substances have been widely used all over the world for various reasons. Alcoholic beverages for example, have played significant social, economic, political, and traditional roles in many civilizations in Europe, America, and Africa [1]. Several other psychoactive substances have been used in societies for one medicinal purpose or the other. Cannabis use for its medicinal properties is believed to have started in China over 4000 years ago [1].

Despite the medicinal benefits of some psychoactive substances and their social acceptability, they are related to some undesirable health, social, legal and economic outcomes [2]. Tobacco accounts for 8.8% (4.9 million) deaths and 4.1% (59.1million) of Disability Adjusted Life Years (DALYs), while illicit drugs such as opioids, 0.4% of deaths and 0.8% of DALYs [2]. Of concern is the increasing relationship between HIV/AIDS infection, violence and substance abuse. In Botswana, heavy use of alcohol has been found to be associated with higher odds of all risky sex behaviors, gender-based violence and HIV transmission in both genders [3]. Furthermore, the relationship between substance use and psychological distress has been demonstrated using the General Health Questionnaire (GHQ) and other psychological instruments [4, 5].

Initiation to substance use mostly starts between the ages of 12 and 24 years and males are more susceptible than females, although this gap narrows as the age of initiation increases [4, 6]. The usual ages of entry into the university especially in this part of the world coincide with the ages of drug initiation, and for some youths, their first contact is during their university education. University life especially the early part has been described as a “transitional” period during which students move from a restricted high school life, mostly supervised by parents, to a more independent life which may be readily influenced by a liberal campus environment [7]. Some other factors that may predispose to drug use among undergraduates include academic pressure, peer pressure, easy accessibility, and unhealthy family background [8]. Alcohol and tobacco are often the first to be initiated of all the psychoactive substances [6, 9]. Alcohol is the most widely used psychoactive substances across the globe, accounting for 90.8% [9]. Except for the United States of America, Brazil, Mexico, Denmark and Spain where cannabis use ranked second, tobacco is the second most commonly consumed drug in most countries [9]. Studies conducted among university undergraduates from different parts of Africa gave similar results [4, 10].

Previous studies conducted in Botswana have only reported on the use of alcohol and tobacco [11–13], which may lead to an erroneous assumption that these are the only drugs abused. Nonetheless, the fact that tobacco has been regarded as a gateway drug [14] suggests the existence of use of other unreported psychoactive substances. To explore this, we set out to investigate the pattern of substance use, its association with psychological distress, and common socio-demographic factors among undergraduates, using an instrument that measures a broader range of drugs. This study will not only add to the existing knowledge and provide a broader picture of substance use but should also identify areas for further research on drug use in Botswana.

## Methods

The study was a cross-sectional descriptive study which assessed substance use among full-time first-year students of a tertiary institution in Botswana. The minimum sample size required was 373, but the research instruments were administered to 410 students based on the calculated minimum sample size with an additional 10% allowance for non-response.

The sampling method and procedure for distribution of questionnaires involved a multi-stage sampling technique (i.e., selection done in stages from faculties through departments until the final sampling units were arrived at). In the first stage, five faculties out of the existing seven faculties were selected by simple random technique (balloting). In the second stage, eight departments were randomly selected from 5 faculties, with one department each from two faculties and two from the remaining three selected faculties which have more departments. In the third stage, about 51 respondents were selected from first-year students in each of the preselected departments.

This study was conducted after obtaining ethical approval from the University of Botswana Research and Ethical Review Committee. The approval to embark on the study was given based on the assurance that the name of the study site (institution) would not be used in the publication. A written informed consent was also obtained from everyone who agreed to participate in the study.

Two research instruments were used for the present study. The first consisted of a modified version of the 37 item *World Health Organization (WHO) drug questionnaire*. The WHO developed the prototype in conjunction with the United Nations Division of Narcotics; the International Narcotics Control Board and the International Council on Alcohol and Addictions. It was designed to aid in epidemiological data collection on drug abuse across different geographical regions of the world, and it is recommended for use among students and other populations. Items in the questionnaire consist of different types of psychoactive/illicit substances, such as Cannabis, cocaine, hallucinogens, opioids, and sedatives. Specific examples relevant to this environment were given for the classes of drugs for simplicity. For example, drugs such as methylphenidate (Ritalin), khat, and crystal-meth were cited in the case of amphetamine-type stimulants (ATS). Participants were also asked to include other forms of drugs not listed in the examples. It measures the lifetime, 12-month and current use (i.e., in the last 30 days) of these psychoactive substances. It also consists of relevant questions on the socio-demographic characteristics of the respondents such as age, religious participation, ethnicity, position in the family, parents’ marital status, parents’ level of education and occupation. Religious participation was measured by frequency of attendance of religious activities, where a subjective response of “never” or “rarely” were grouped as poor participation and “regularly” as good participation. A fictional drug ‘maladrine’ was added to the list of psychoactive substances. Those who agreed to have taken this fictional substance were excluded from further analysis to reduce the bias of over-reporting.

The second instrument comprised of the *12-item General Health Questionnaire (GHQ − 12*), was used to assess the presence of psychological distress in the students. The GHQ-12 is a 12 item screening device for identifying minor psychiatric disorders in the general population and is suitable for adolescents and young adults. It has been found to have good internal consistency across many cultures as reflected by Cronbach’s alpha range from 0.82–0.89 in regions as diverse as Africa, Asia, Europe and South-America [15–17]. Each question has four possible responses; the respondent was asked to choose only one response which best fits how he/she felt recently. The first two responses were scored “0” while the last two were scored “1” each. A score of 1 on each item was considered positive and a score of 0, negative. Positive scores were indicative of psychological distress. The cut-off of 3 was derived from the calculated GHQ mean score; hence, a score of 3 or more was used in this study to indicate psychological distress as in a previous study in a similar setting [4].

Data Analysis was done using the Statistical Package for Social Sciences (SPSS for Windows), Version 16. Frequency tables were employed for descriptive statistics such as the socio-demographic variables and prevalence of drug use. Cross-tabulations were done to show the prevalence of substance use by gender, and the relationships between substance use and GHQ score. A bivariate analysis was performed to explore the relationship between identified socio-demographics and current use of any substance. To further explore this relationship, the significant variables on bivariate analysis were entered into a binary logistic regression, with current use of any substance as the dependent variable. Fishers Exact Test (FET) was used where applicable. The level of statistical significance for all tests was set at *p* < 0.05.

## Results

### Socio-demographic characteristics of the respondents

Out of 410 students interviewed, only 401 (97.8%) responses were analyzed. The remaining nine were excluded for admitting to the use of fictional drug, ‘maladrine,’ that was intentionally included to forestall over-reporting, and for incomplete responses. The mean age of the respondents was 20.8 (SD =1.4) years, while the age range was 18–24 years. More female students (50.4%) participated in the study than males (49.6%). Over half (58%) of the participants came from the Tswana ethnic group. Christianity was the most predominant religion (63.6%), followed by African traditional religion (18.9%), and others (8.8%), such as Hinduism and Buddhism. More than two-thirds of the respondents participated regularly in religious activities and received monthly allowance below150 USD (Table 1).

**Table 1:** Socio-demographic characteristics of the respondents

Variable	Statistic	
Age, years; mean (sd)	20.8 (1.4)	
Age range	18–24 years	
	Frequency N	Percent
Age group^a^	391	100
< 21 years	219	56.0
21–25 years	164	41.9
> 25	8	2.0
Gender	401	100
Male	199	49.6
Female	202	50.4
Religion^a^	396	100
Christianity	252	63.6
Islam	11	2.8
Traditional religion	75	18.9
Others	35	8.8
No religious affiliation	23	5.8
Religious participation	389	100
Rare or low religious participation	89	22.9
Regular participation	300	77.1
Ethnicity	393	100
Tswana	229	58.3
Kalanga	122	31.0
Others	15	3.9
Foreigners	27	6.9
Monthly Upkeeps^a^	347	100
Below 130 USD	97	28.0
130–150 USD	199	57.3
Above 150 USD	51	14.7

^a^N = n not equal to 401 due to missing data

### Prevalence and pattern of drug use

**Fig. 1:**
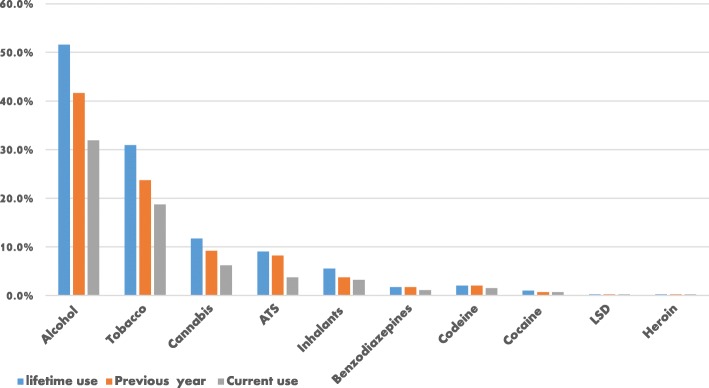
Showing the prevalence of psychoactive substance use among respondents. LSD - Lysergic acid diethylamide. ATS - Amphetamine-type stimulants

The lifetime prevalence of any substance use (defined as at least a single episode of use) was 59.6%, 12-months prevalence (previous year) was 49.4% while the current use was 37.9%. The lifetime prevalence of multiple drug use, technically defined as the use of more than one psychoactive substance, was 45.1%, 12-months (previous year) was 42.4% and current use 36.4%. Alcohol was the most commonly used psychoactive substance with 31.9% current users. Of these, beer (51.5%) and wine (26.6%) were the most frequent types. This was followed by tobacco which was 18.7% and cannabis, 6.2% (Fig. 1). Inhalants, which were majorly in the form of petrol and glue, were 3.2%. ATS, which comprised of methylphenidate, street drugs, crystal meth, and khat were 3.7%, while controlled drug such as benzodiazepines was 1.1%. Except for inhalants (FET, *p* < 0.01), no gender difference was observed in the rates of psychoactive substance use. Majority of the psychoactive substances were first tried between the ages of 15–18 years. None of the illegal drugs (cocaine, cannabis, heroin, and codeine) uses started before the age of 11 years, but most of those who use inhalants or solvents such as petrol started at the age of 10 (Table 2).

**Table 2:** Age at first use of drugs among respondents

Age at first drug use in years, n (%)					
Drugs	Frequency (N)	10 or less	11–14	15–18	19 or more
Tobacco	120	6 (5.0)	24 (20.0)	54 (45.0)	36 (30.0)
Alcohol	200	14 (7)	17 (8.5)	93 (46.5)	76 (38.0)
Cannabis	45	–	3 (6.7)	20 (44.4)	22 (48.9)
Cocaine	4	–	–	3 (75.0)	1 (25.0)
ATS	33	5 (15.2)	2 (6.1)	8 (24.2)	18 (54.5)
Inhalants	17	11 (64.7)	3 (17.6)	1 (5.9)	2 (11.8)
Benzodiazepines	6	1 (16.7)	1 (16.7)	1 (16.7)	3 (50.0)
Heroin	1	–	1 (100)	–	–
Codeine	6	–	1 (16.7)	3 (50.0)	2 (33.3)
LSD	1	–	–	–	1 (100)

*LSD* lysergic acid diethylamide, *ATS* Amphetamine-type stimulants

### Drug use and psychological distress

About 16% of the students had significant psychological distress, (defined as GHQ score of 3 and above). Current use of alcohol (χ^2^ = 4.03, *p* = 0.045), ATS (χ^2^ = 8.35, *p* = 0.004) and benzodiazepines (FET, *p* = 0.021) were associated with significant psychological distress. Similarly, lifetime use of benzodiazepines (FET, 0.005) and codeine (FET, 0.045) were associated with GHQ of 3 and above (Table 3).

**Table 3:** Respondents’ drug use and GHQ score

Drugs	GHQ score			
	GHQ (0–2) N (%)	GHQ (3–12) N (%)	χ^2^	*p*
Tobacco				
Current use	60 (87.0)	9 (13.0)	1.64	0.200
Alcohol				
Current use	104 (87.4)	15 (12.6)	4.03	*0.045*
Cannabis				
Current use	17 (70.8)	7 (29.2)	1.99	0.158
Cocaine				
Current use	3 (100)	–		0.99
ATS				
Current use	8 (53.3)	7 (46.7)	8.35	*0.004*
Inhalants				
Current use	11 (91.7)	1 (8.3)	0.82	0.360
Benzodiazepines				
Current use	1 (25.0)	3 (75.0)		*0.021* ^*^

*ATS* amphetamine-type stimulants

^*^FET = Fisher’s Exact Test. Significant *p*- value in *italics*

### Factors that were associated with any substance use among university undergraduates

Factors that were significant on bivariate analysis were age 20 years and below (χ^2^ = 7.34, *p* = 0.007), poor participation in religious activities (χ^2^ = 33.8, *p* < 0.01), earning 150 USD and above per month (χ^2^ = 6.20, *p* = 0.013), having a father who smokes cigarette (χ^2^ = 6.81, *p* = 0.009), having a father who drinks alcohol (χ^2^ = 19.5, *p* = < 0.01), and having a friend who uses drugs (χ^2^ = 6.57, *p* = 0.010) (Table 4). With logistic regression analysis, only two of these variables were found to have significant relationships with any substance use. Poor participation in religious activities was positively related with any substance use (OR = 4.63, 95%CI: 2.03–10.51), not having a friend who uses drugs was negatively associated with any substance use (OR = 0.44, 95%CI: 0.19–0.99) (Table 5).

**Table 4:** Bivariate analysis showing the relationship between identified risk factors and any substance use

Variables	Any Substance use		Statistic		
	No	yes			
	N(%)	N(%)	df	χ^2^	*P*
Age (years)	149 (68.0)	70 (32.0)	1	7.34	*0.007*
20 and below	94 (54.7)	78 (45.3)			
Above 20					
Ethnicity					
Tswana	143 (62.4)	86 (37.6)	4	7.46	0.059
Kalanga	78 (63.9)	44 (36.1)			
Others	12 (80.0)	3 (20.0)			
Non-citizens	11 (40.0)	16 (59.3)			
Religion					
No religious affiliation	15 (65.2)	8 (34.0)	4	1.18	0.881
Christianity	152 (60.3)	100 (39.7)			
Islamic religion	7 (63.6)	4 (36.4)			
African traditional religion	48 (64.0)	27 (36.0			
Others	24 (68.6)	11 (31.4)			
Religious participation					
Rare or low religious participation	32 (36.0)	57 (64.0)	1	33.8	*< 0.01*
Regular participation	210 (70.0)	90 (30.0)			
Upkeep/month					
Below 150 USD	71 (73.2)	26 (26.8)	1	6.20	*0.013*
150 USD and above	147 (58)	103 (41.2)			
Father’s level of education					
Below secondary school	45 (67.2)	22 (32.8)	1	0.104	0.747
Secondary school and above	130 (65.0)	70 (35.0)			
Mother’s level of education					
Below secondary school	52 (58.4)	37 (41.6)	1	0.68	0.409
Secondary school and above	181 (63.3)	105 (36.7)			
Father’s employment status					
No employement	43 (63.2)	25 (36.8)	1	0.043	0.835
Employed	137 (64.6)	75 (35.4)			
Marital status of the parents					
Divorced	46 (58.2)	33 (41.8)	2	2.13	0.350
Married and staying together	112 (66.7)	56 (33.3)			
Separated or never married	71 (60.2)	47 (39.8)			
Father smoking cigarette					
No	112 (72.7)	42 (27.3)	1	6.81	*0.009*
Yes	52 (56.5)	40 (43.5)			
Father drinking alcohol					
No	91 (77.8)	26 (22.2)	1	19.5	*< 0.01*
Yes	71 (51.1)	68 (48.9)			
Mother smoking cigarette					
No	218 (62.5)	131 (37.5)	1	0.28	0.594
Yes	6 (54.5)	5 (45.5)			
Mother drinking alcohol					
No	203 (63.6)	116 (36.4)	1	0.78	0.378
Yes	22 (56.4)	17 (43.6)			
Friend using any substance					
No	200 (64.3)	111 (35.7)	1	6.57	*0.010*
Yes	34 (47.9)	37 (52.1)			

Significant *p*-value in *italics*

**Table 5:** Logistic regression showing the factors that are associated with substance use among students

Variables	Statistics				
				95% Confidence Interval	
	Wald	*p*-value	OR	lower	upper
Age (20 years and below)	0.10	0.757	1.11	0.57	2.19
Rare or low religious participation	13.4	*< 0.001*	4.63	2.03	10.51
Earn below 150 USD per month	0.04	0.851	0.93	0.43	1.99
Father does not smoke cigarette	0.53	0.467	0.75	0.35	1.61
Father does not drink alcohol	2.25	0.134	0.55	0.25	1.20
Does not have a friend abusing drug	3.96	*0.047*	0.44	0.19	0.99

Significant *p*-value in *italics*

## Discussion

Drug use among undergraduates is as noteworthy a problem in Botswana as elsewhere [8, 18–20] and has mainly been underreported. The present study, to our knowledge, was the first to determine the pattern of general drug use among university students in Botswana. The lifetime prevalence for any substance use was 59.6%; 12-month prevalence was 49.4% while the current use was 37.9%. These figures are within the range described in previous literature from the developed countries [9, 20, 21] and are similar to those found in Africa as well. For example in West Africa, the lifetime prevalence of any substance use ranged from 56 to 78% and 1-month prevalence, 28–40% among undergraduates [4, 8]. There was a high prevalence of multiple drug use among the respondents with 36.4% currently engaging in multiple drug use. Multiple drug use has been associated with higher rates of complications including rule-breaking behavior [22], sexual and physical abuse [3], and various other psychiatric disorders [5]. This relationship is complex and multidirectional. While this present study did not set out to assess the relationships between drug use and anti-social or high-risk behaviors, the finding of a high prevalence of multiple drug use may be an indication that this population is a high-risk group for complications of drug abuse.

As in many other studies on drug use in youths [4, 9, 20], alcohol was the most commonly used psychoactive substance with a current prevalence of 31.9%. The significant role of alcohol in many social functions, its wide availability and the social acceptability of its use, are reasons that have been adduced for this trend [8]. Production of alcohol (mostly from Sorghum) and its consumption has been an integral part of the culture and village life in Botswana, but consumption of alcoholic beverages was traditionally restricted to elders of the community [23]. Rapid industrialization and economic globalization have brought about sudden and extensive social transformations in the developing countries, including Botswana. Children and women who were previously excluded from drinking now constitute a significant proportion of consumers of these products [23]. However, our study revealed a lower prevalence than what was previously reported in Botswana, 58% [13], 44.4% [12] and Nigeria, 58% [4] among undergraduates. While we cannot make a direct comparison between the current study and the previously conducted studies in Botswana, it is possible that the 30% tax placed on its purchase is playing a significant role in the reduction of alcohol consumption in Botswana, as suggested by previous authors [24].

Tobacco was the next most commonly used psychoactive substance in this study group as reported elsewhere [4, 9, 20] with a current prevalence of 18.7%. This rate is much higher than the prevalence reported in the Global youth tobacco surveillance report from 2000 to 2007 (9.5%) [25] but similar to the rates from Europe (19.2%) and America (15%) [25]. The rate reported in this study is also lower than what was previously reported among teachers in Botswana. Although, the low prevalence among the teachers could have been related to Botswana legislation on tobacco control (Botswana Control of Smoking Act, 1992), which strictly prohibits any direct forms of tobacco advertising and use, it is possible that youths are less likely to follow this law. Moreover, only those who have recently completed their first 1 year on campus were included in the present study, and this may also explain the higher prevalence of tobacco use in this sample. First-year students often face significant challenges such as exposure to new courses, more liberal campus environment, peer pressure, securing accommodation, which may stretch their capacity to cope and engender substance use behavior. Among youths, alcohol and tobacco have been referred to as “soft drugs” taking into consideration that they are legal in most countries and also as “gateway drugs,” based on the trend of being the first psychoactive substance taken by many users before “graduating’ to other psychoactive substances [14]. The respondents in our study seem to maintain that pattern. It is, therefore, necessary to set up policies and programs targeted towards controlling the use of these “soft drugs” and prevent the progression to more dangerous drugs.

Consistent with the findings of the global report, cannabis was the most commonly used illicit substance with 9.2% admitting to using the substance within the last 12 months and 6.2% current users. The rate of those who have used the substance in the last 12 months is similar to what has been reported by previous authors from other countries [4, 10, 19, 21]. Nevertheless, the rates of other illicit and prescription drugs such as cocaine, codeine, ATS, and benzodiazepines are quite low as in other studies from Africa [4, 19], but unlike in the United States, where their uses have been reported to be on the increase [18, 20]. As in other African countries, it is possible that these substances are too expensive to procure and sustain in Botswana, especially by these individuals who mostly survive on stipends from the government.

It is important to note that, apart from inhalant use which was mainly a male-dominated affair, no gender difference was observed in the use of other substances. These findings suggest that drug use is not an entirely male-dominated activity in Botswana. Conversely, gender-based roles prescribed for women are protective against drug abuse behaviors in many communities [4, 23]. Men have been reported to be more likely to use tobacco and other illicit substances than women [4, 20, 22]. Perhaps, the nature of our sample is responsible for this disparity. In Botswana, due to the adoption of western social norms particularly amongst urban youths, there is a little cultural restriction against the use of psychoactive substances by females. This is evidenced by the open consumption of alcohol and tobacco without any apparent social censure. This practice is entirely different from some other regions in Africa, where such habits are deemed socially unacceptable [4, 26].

For most of the substances assessed the age range of debut was 15–18 years. This finding may indicate a critical window period for drug abuse prevention programs in the population. The substances which bucked this trend included solvents such as petrol (aged 10 or less), alongside ATS and benzodiazepines with a higher age of debut (19 and above). As it has been previously documented, this finding suggests that inhalants may play a role as a gateway drug as tobacco [27], and may require specific attention in any drug abuse prevention programs directed toward the pre-adolescent age group. The drugs which had a relatively lower age of debut, i.e., alcohol, tobacco, and inhalants are readily available in the community. Alcohol and inhalants are even more easily accessible for the pre-adolescents since alcohol is freely available in many African households and inhalants such as glue can be purchased without restrictions. Our finding demonstrates that universal preventive techniques that result in reduced availability and accessibility of such substances may likely result in reduced consumption and abuse of the substances. In addition to the tax and restriction of alcohol and cigarette sales to under-18’s, parents should also be educated on how to prevent access to these substances at home. Perhaps there should also be a restriction on sales of inhalants to children less than 18 years, as in alcohol and tobacco. There is increasing evidence suggesting that a lower age of debut is associated with a higher rate of problems with drug use ranging from dependence, other drug-related disorders to delinquency [6], and other mental disorders [5]. Thus, no effort should be spared in preventing minors from experimenting with psychoactive substances.

Current use of either alcohol or ATS was seen to be significantly associated with psychological distress in the current study. It is imperative to note that, the direction of the correlation cannot be assumed due to the cross-sectional nature of this study. Perhaps, the psychological distress contributed to or led to substance use, and not vice-versa. This assumption deserves further investigation. Even so, it has been established that despite the social acceptability of alcohol use, it still presents a public health concern. It is estimated that about 4% of all deaths worldwide are attributable to alcohol consumption along with 4.5% of the global disease and injury burden [28]. For example in Botswana, alcohol-impaired road traffic crashes are one of the leading causes of disabilities and deaths [24]. The impact of alcohol consumption on public health statistics cannot be overstated. There is a need to reduce alcohol accessibility and availability to adolescents. There may also be the need to make policies on the attractiveness of the packaging and to limit advertisements of alcoholic products similarly as it is being done for tobacco. The use of benzodiazepines and codeine were also significantly associated with psychological distress, albeit these findings should be interpreted with caution due to the sample size. These “Central Nervous System depressants” are in most cases prescription drugs diverted for recreational purposes through self-medication. These drugs are already highly regulated. There is, therefore, a need to enforce the existing regulations and improve them where necessary.

On bivariate analysis, participants whose fathers were not using any psychoactive substance (alcohol or tobacco), and those who live on less than 150 USD per month were less likely to use drugs. Studies have shown that parental substance use is a factor in substance use among their offspring [29]. For example, Kilpatrick et al. found an increased risk of substance abuse in adolescents who had family members with alcohol or drug use problem [30]. In the same vein, smoking in adolescents has been found to be associated with lower family socioeconomic status [31]. Cigarettes are cheaper and more accessible for students with little financial resources. Despite these finding, these variables, including age groups failed to predict psychoactive substance use in our sample on logistic regression.

Logistic regression showed that poor participation in religious activities was positively correlated with substance use while not having a friend who uses drugs was negatively correlated. This finding has been shown in studies elsewhere [32, 33]. In a study conducted among 12595 Brazilian university students, frequent participation in religious activities was shown to have a protective effect against substance use [32]. It is possible that participation in religious activities provides opportunities to interact with non-drug using peers. Since peer influence is at peak during adolescence [34], the negative correlation between not having a friend who uses drugs and substance use is thus not surprising. Peer group influence has been shown to be a factor in substance use [35] whereas, religious students have also been noted to be less prone to engage in risky behavior [36] such as drug abuse. How to harness these seemingly protective influences is a field for further study.

## Conclusions

There is evidence that the use of psychoactive substances is a problem in the undergraduate population in Botswana. The most commonly used psychoactive substance was alcohol as elsewhere, but the government 30% tax policy on alcoholic beverages may have contributed to the relatively lower rate in the current study. There is a high rate of the early debut of inhalants, tobacco, and alcohol, which is possibly driven by their availability to pre-adolescents mainly. Poor participation in religious activities was positively correlated with substance use while not having a friend who uses drugs was negatively correlated. The role of religious participation in addressing drug abuse on campus should be further explored.

### Recommendations

This study has demonstrated that there is a significant problem of drug use amongst youths in Botswana and it suggests a need for urgent action to reverse the trend. Proper orientation package should be designed for fresh students to enable them to adjust and adapt to the new stage of life very easily. School health policies should be adjusted to include programmes targeted towards drug education and counseling. Participation in religious activities appears to plays an important role in inhibiting the use of a psychoactive substance. Therefore, a conducive environment which encourages religious activities, and other adaptive ways of relieving stress should be encouraged in the university community.

### Limitations and strengths

The study was conducted in one tertiary institution and may not be generalizable to the entire adolescent population of Botswana, particularly in the context of relatively low university enrolment. It was also cross-sectional descriptive and may not be able to determine causality of any of its findings. It is, however, the first to look at substance use generally and not a specific or few substances. It also attempted to see a relationship between substance use and the associated problems of psychological distress.

### Future research

It will be necessary to conduct a similar study in the general youth population to see how the rates in this group compare with the general youth population. It will be helpful to assess the relationships between substance use and other associated problems like criminal offending and academic performance within this group and other similar groups. The role of religious participation in addressing drug abuse on campus should be further explored. There is also a need to conduct a prospective study on drug users particularly those with a lower age of debut to assess the relationship of age of debut with outcomes.
